# Middle East Respiratory Syndrome Coronavirus Antibodies in Bactrian and Hybrid Camels from Dubai

**DOI:** 10.1128/mSphere.00898-19

**Published:** 2020-01-22

**Authors:** Susanna K. P. Lau, Kenneth S. M. Li, Hayes K. H. Luk, Zirong He, Jade L. L. Teng, Kwok-Yung Yuen, Ulrich Wernery, Patrick C. Y. Woo

**Affiliations:** a State Key Laboratory of Emerging Infectious Diseases, The University of Hong Kong, Hong Kong; b Carol Yu Centre for Infection, The University of Hong Kong, Hong Kong; c Collaborative Innovation Centre for Diagnosis and Treatment of Infectious Diseases, The University of Hong Kong, Hong Kong; d Department of Microbiology, Li Ka Shing Faculty of Medicine, The University of Hong Kong, Hong Kong; e Central Veterinary Research Laboratory, Dubai, United Arab Emirates; University of Maryland, College Park

**Keywords:** Bactrian camel, hybrid camel, MERS coronavirus, antibody

## Abstract

So far, dromedary camels are the only known animal reservoir for Middle East respiratory syndrome (MERS) coronavirus (MERS-CoV). Previous published serological studies showed that sera of Bactrian camels were all negative for MERS-CoV antibodies. However, a recent study revealed that direct inoculation of Bactrian camels intranasally with MERS-CoV can lead to infection with abundant virus shedding and seroconversion. In this study, we examined the presence of MERS-CoV antibodies in Bactrian and hybrid camels in Dubai, the United Arab Emirates (where dromedaries are also present), and Bactrian camels in Xinjiang, China (where dromedaries are absent). For the 29 serum samples from Bactrian camels in Dubai tested by the MERS-CoV spike (S) protein-based enzyme-linked immunosorbent assay (S-ELISA) and neutralization antibody test, 14 (48%) and 12 (41%), respectively, were positive for MERS-CoV antibodies. All the 12 serum samples that were positive with the neutralization antibody test were also positive for the S-ELISA. For the 11 sera from hybrid camels in Dubai tested with the S-ELISA and neutralization antibody test, 6 (55%) and 9 (82%), respectively, were positive for MERS-CoV antibodies. All the 6 serum samples that were positive for the S-ELISA were also positive with the neutralization antibody test. There was a strong correlation between the antibody levels detected by S-ELISA and neutralizing antibody titers, with a Spearman coefficient of 0.6262 (*P* < 0.0001; 95% confidence interval, 0.5062 to 0.7225). All 92 Bactrian camel serum samples from Xinjiang were negative for MERS-CoV antibodies tested using both S-ELISA and the neutralization antibody test. Bactrian and hybrid camels are potential sources of MERS-CoV infection.

**IMPORTANCE** Since its first appearance in 2012, Middle East respiratory syndrome (MERS) has affected >25 countries, with >2,400 cases and an extremely high fatality rate of >30%. The total number of mortalities due to MERS is already greater than that due to severe acute respiratory syndrome. MERS coronavirus (MERS-CoV) has been confirmed to be the etiological agent. So far, dromedaries are the only known animal reservoir for MERS-CoV. Previously published serological studies showed that sera of Bactrian camels were all negative for MERS-CoV antibodies. In this study, we observed that 41% of the Bactrian camel sera and 55% of the hybrid camel sera from Dubai (where dromedaries are also present), but none of the sera from Bactrian camels in Xinjiang (where dromedaries are absent), were positive for MERS-CoV antibodies. Based on these results, we conclude that in addition to dromedaries, Bactrian and hybrid camels are also potential sources of MERS-CoV infection.

## Abstract (precis)

Since its first appearance in 2012, Middle East respiratory syndrome (MERS) has affected >25 countries, with >2,400 cases and an extremely high fatality rate of >30%. The total number of mortalities due to MERS is already greater than that due to severe acute respiratory syndrome. MERS coronavirus (MERS-CoV) has been confirmed to be the etiological agent. So far, dromedaries are the only known animal reservoir for MERS-CoV. Previously published serological studies showed that sera of Bactrian camels were all negative for MERS-CoV antibodies. In this study, we observed that 41% of the Bactrian camel sera and 55% of the hybrid camel sera from Dubai (where dromedaries are also present), but none of the sera from Bactrian camels in Xinjiang (where dromedaries are absent), were positive for MERS-CoV antibodies. Based on these results, we conclude that in addition to dromedaries, Bactrian and hybrid camels are also potential sources of MERS-CoV infection.

## OBSERVATION

Since its first appearance in 2012, Middle East respiratory syndrome (MERS) has affected more than 25 countries in 4 continents, with more than 2,400 cases and an extremely high fatality rate of more than 30%. The total number of mortalities due to MERS is already greater than that due to severe acute respiratory syndrome. MERS coronavirus (MERS-CoV), a betacoronavirus from subgenus *Merbecovirus*, has been confirmed to be the etiological agent (1). Human dipeptidyl peptidase 4 was found to be the cellular receptor for MERS-CoV (2). Subsequent detection of MERS-CoV and its antibodies in dromedary, or one-humped, camels (Camelus dromedarius) in various countries in the Middle East and North Africa have suggested that these animals are probably the reservoir for MERS-CoV (3–5). Other betacoronaviruses in bats from the subgenus *Merbecovirus* (e.g., *Tylonycteris* bat CoV HKU4, *Pipistrellus* bat CoV HKU5, *Hypsugo* bat CoV HKU25), and hedgehogs were found to be closely related to MERS-CoV (6–9).

**FIG 1:**
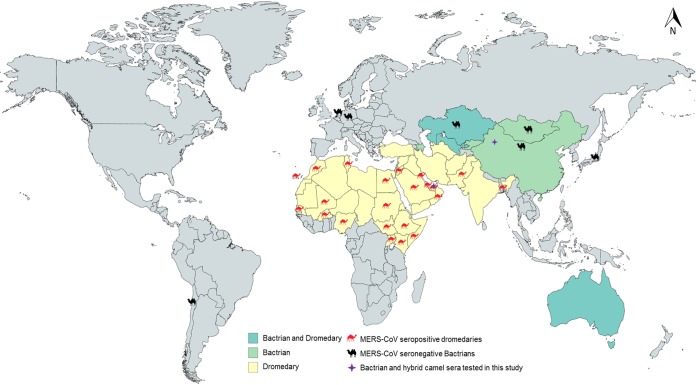
Geographical distribution of dromedaries and Bactrians. Places with MERS-CoV-seropositive dromedaries (red camels) and MERS-CoV-seronegative Bactrians (black camels) from previous studies are labeled.

In addition to the dromedaries, there are two additional surviving Old World camels, the Bactrian, or two-humped, camels (Camelus bactrianus) and the wild Bactrian camels (Camelus ferus), both inhabitants of Central Asia. Moreover, a dromedary and a Bactrian camel can mate and result in a hybrid camel offspring. Previous published serological studies showed that sera of Bactrian camels were all negative for MERS-CoV antibodies, suggesting that Bactrian camels may not be a reservoir of MERS-CoV (Fig. 1) (10–14). However, a recent study revealed that direct inoculation of Bactrian camels intranasally with MERS-CoV can lead to infection with abundant virus shedding and seroconversion (15). Therefore, we hypothesize that those Bactrian camels, and even the hybrid camels, that reside in countries where there are dromedaries can be infected with MERS-CoV. To test this hypothesis, we examined the presence of MERS-CoV antibodies in Bactrian and hybrid camels in Dubai, the United Arab Emirates (where dromedaries are also present), and Bactrian camels in Xinjiang, China (where dromedaries are absent), using a MERS-CoV spike (S) protein-based enzyme-linked immunosorbent assay (ELISA) and neutralization antibody test.

A total of 29 and 11 serum samples, respectively, were collected from 29 Bactrian camels and 11 hybrid camels from a private collection in Dubai (April to May 2019) (Table 1), and 92 serum samples were collected from Bactrian camels on a camel farm in Xinjiang (November 2012) (16). Antibodies against the S protein of MERS-CoV were tested using microplates precoated with purified (His)_6_-tagged recombinant receptor-binding domain of S (RBD-S) of MERS-CoV and detected with 1:8,000 diluted horseradish peroxidase-conjugated goat anti-llama IgG (Life Technologies, Carlsbad, CA, USA) conjugate (S-ELISA) (17). The cutoff of the ELISA was defined as three standard deviations above the mean absorbance value of 10 dromedary serum samples that tested negative with the neutralization antibody test. The neutralization antibody test was performed by incubating serially diluted camel sera with 100 50% tissue culture infective dose (TCID_50_) MERS-CoV for 2 hours before infecting the Vero cells for 1 hour. The cytopathic effect (CPE) was observed for 5 days, and the sera were regarded as positive for neutralizing antibody if no CPE was observed in the infected cells (18). All tests were performed in triplicate.

**TABLE 1:** MERS-CoV neutralizing antibody titer of Bactrian and hybrid camel sera

Neutralizing antibody titer	No. of samples (%) for:		
	Dubai Bactrian camel (*n* = 29)	Dubai hybrid camel (*n* = 11)	Xinjiang Bactrian camel (*n* = 92)
<10	17 (58.6)	2 (18.2)	92 (100)
10	0 (0)	0 (0)	0 (0)
20	0 (0)	0 (0)	0 (0)
40	0 (0)	0 (0)	0 (0)
80	0 (0)	0 (0)	0 (0)
160	1 (3.4)	1 (9.1)	0 (0)
320	2 (6.9)	2 (18.2)	0 (0)
640	9 (31.1)	6 (54.5)	0 (0)

**FIG 2:**
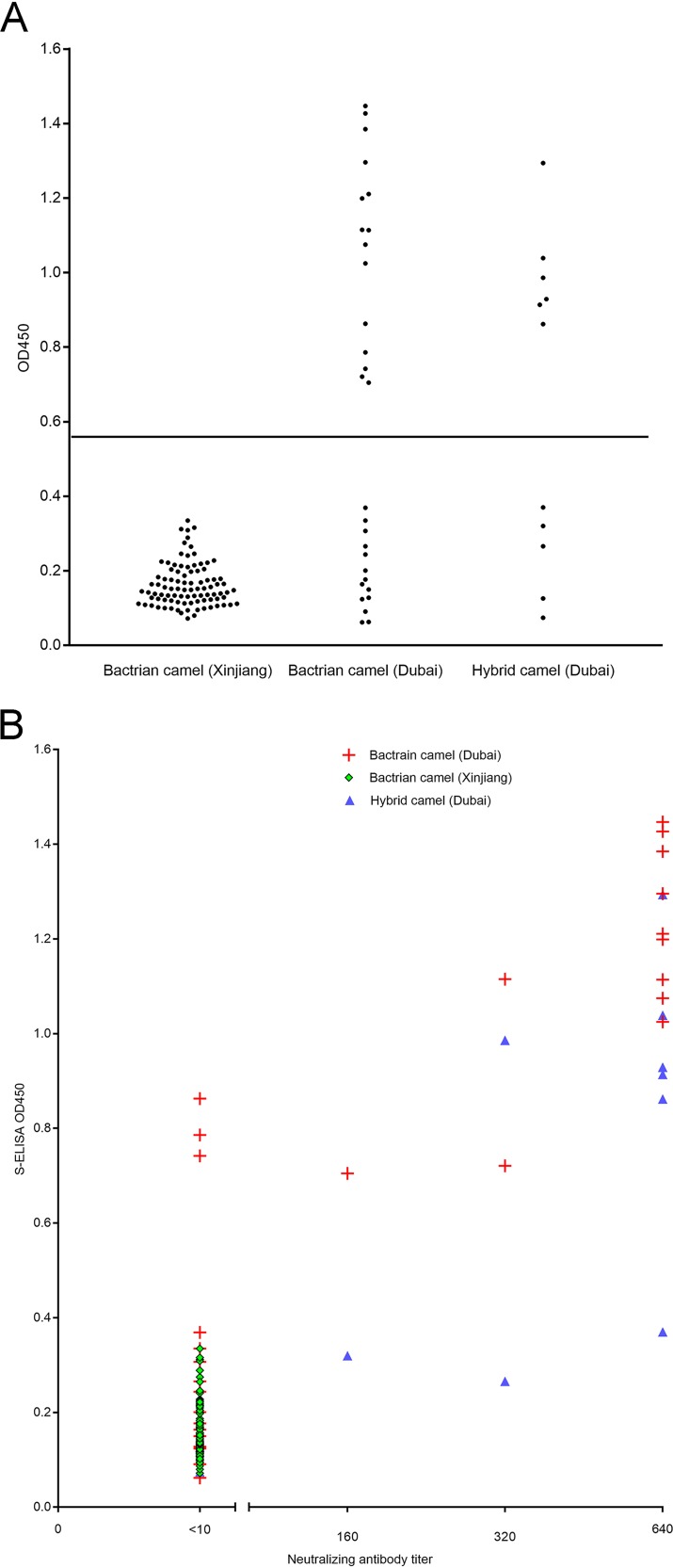
(A) Scatter plot showing MERS-CoV antibody levels detected using S-ELISA in Bactrian and hybrid camel sera from Dubai and Xinjiang. The test results were plotted as optical density at 450 nm (OD_450_) values. The horizontal line indicates the cutoff value (0.557) for positive diagnosis. (B) Scatter plot showing correlation between antibody levels detected using S-ELISA and neutralizing antibody titers of Bactrian and hybrid camel sera for MERS-CoV.

For the 29 serum samples from Bactrian camels in Dubai tested with the S-ELISA and neutralization antibody test, 14 (48%) and 12 (41%), respectively, were positive for MERS-CoV antibodies (Fig. 2A and Table 1). All the 12 serum samples that were positive with the neutralization antibody test were also positive for the S-ELISA. For the 11 serum samples from hybrid camels in Dubai tested with the S-ELISA and neutralization antibody test, 6 (55%) and 9 (82%), respectively, were positive for MERS-CoV antibodies (Fig. 2A and Table 1). All the 6 serum samples that were positive for the S-ELISA were also positive with the neutralization antibody test. There was a strong correlation between the antibody levels detected by S-ELISA and neutralizing antibody titers, with a Spearman coefficient of 0.6262 (*P* < 0.0001; 95% confidence interval, 0.5062 to 0.7225) (Fig. 2B). All 92 Bactrian camel serum samples from Xinjiang were negative for MERS-CoV antibodies tested with both S-ELISA and the neutralization antibody test (Fig. 2A and Table 1).

We showed that MERS-CoV antibodies were present in Bactrian and hybrid camels from Dubai. When the reservoir of MERS-CoV was first discovered in dromedaries (3), it was wondered whether Bactrian camels are also another possible source of the virus. However, in all the studies that looked for MERS-CoV in both wild (China, *n* = 190; Kazakhstan, *n* = 95; Mongolia, *n* = 200) and captive (the Netherlands, *n* = 2; Chile, *n* = 2; Japan, *n* = 5; Germany, *n* = 16) Bactrian camels, it was found that none of the Bactrian camels, as tested with neutralization antibody test, ELISA, immunofluorescence assay, plaque reduction neutralization test, and/or protein microarray, had MERS-CoV antibodies (10–14). However, the sera from all these studies were obtained from Bactrian camels in geographical regions where there were no dromedaries that were positive for MERS-CoV or its antibodies. Interestingly, in a recent study that investigated the susceptibility of Bactrian camels to MERS-CoV, upon intranasal inoculation with 10^7^ TCID_50_ of MERS-CoV, the Bactrian camels developed clinical signs of transient upper respiratory tract infections, such as nasal discharge and coughing, with shedding of up to 106.5 to 106.8 plaque forming units/ml of MERS-CoV from the upper respiratory tract and development of neutralizing antibodies against MERS-CoV (15). This suggested that Bactrian camels can be susceptible to MERS-CoV infection and may actually be another possible reservoir for MERS-CoV. In the present study, we showed that MERS-CoV antibodies were present in 41% of the Bactrian camels and 55% of the hybrid camels from a private collection in Dubai using two independent assays. These camels were kept as hobby animals. They had occasional contacts with dromedaries from camel farms that breed dromedaries for racing. MERS-CoV has been consistently detected in these camel farms in the last few years, and the MERS-CoV seropositive rate increased as the age of the dromedaries increased (17). The Bactrian camels were imported from Kazakhstan more than 10 years ago, whereas the hybrid camels were the offspring of mating between dromedaries and the Bactrian camels. Some of the hybrid camels were sometimes used for camel racing and therefore had more frequent contacts with dromedaries. It is likely that some of the Bactrian and hybrid camels might have acquired the MERS-CoV during their contacts with dromedaries that were shedding the virus, and the virus subsequently infected other Bactrian and hybrid camels in the collection.

To prevent MERS in humans, it might be worthwhile to immunize Bactrian and hybrid camels in addition to the dromedaries. So far, as determined from the results of phylogenomic analyses, several clades of MERS-CoV are circulating in dromedaries. Although it seems that the ultimate origin of MERS-CoV was from bats (6), there are still significant differences between the genome sequences of these betacoronaviruses in bats from the subgenus *Merbecovirus* and MERS-CoV, suggesting that interspecies jumping from bats to camels may not be a very recent event, and hence the dromedaries are probably the reservoir of MERS-CoV where the virus was transmitted to humans. In the last few years, a number of MERS-CoV vaccines have been developed for their potential use in dromedaries (19–23). In this study, our results indicated that Bactrian and hybrid camels are also potential sources of MERS-CoV infection. Therefore, Bactrian and hybrid camels, in addition to the dromedaries, should be immunized in order to reduce the chance of transmitting the virus to humans.
